# An ALS-associated variant of the autophagy receptor SQSTM1/p62 reprograms binding selectivity toward the autophagy-related hATG8 proteins

**DOI:** 10.1016/j.jbc.2021.101514

**Published:** 2021-12-18

**Authors:** Andrew Brennan, Robert Layfield, Jed Long, Huw E.L. Williams, Neil J. Oldham, Daniel Scott, Mark S. Searle

**Affiliations:** 1 Centre for Biomolecular Sciences, School of Chemistry, University Park, University of Nottingham, Nottingham, UK; 2 School of Life Sciences, University of Nottingham Medical School, Nottingham, UK; 3 School of Chemistry, University Park, University of Nottingham, Nottingham, UK

**Keywords:** SQSTM1/p62, AIM, autophagy, amyotrophic lateral sclerosis, hATG8, AIM, ATG8-interacting motif, ALS, amyotrophic lateral sclerosis, CSP, chemical shift perturbation, ESI-MS, electrospray ionization–mass spectrometry, FTLD, frontotemporal lobar degeneration, GABARAP, γ-aminobutyric acid receptor associated protein, hATG8, human autophagy-related 8, ITC, isothermal titration calorimetry, LC3, microtubule-associated protein 1A/1B light chain 3, NMR, nuclear magnetic resonance, p62, SQSTM1/p62, UBA, ubiquitin-associated

## Abstract

Recognition of human autophagy-related 8 (hATG8) proteins by autophagy receptors represents a critical step within this cellular quality control system. Autophagy impairment is known to be a pathogenic mechanism in the motor neuron disorder amyotrophic lateral sclerosis (ALS). Overlapping but specific roles of hATG8 proteins belonging to the LC3 and GABARAP subfamilies are incompletely understood, and binding selectivity is typically overlooked. We previously showed that an ALS-associated variant of the SQSTM1/p62 (p62) autophagy receptor bearing an L341V mutation within its ATG8-interacting motif (AIM) impairs recognition of LC3B *in vitro*, yielding an autophagy-deficient phenotype. Improvements in understanding of hATG8 recognition by AIMs now distinguish LC3-interaction and GABARAP-interaction motifs and predict the effects of L341V substitution may extend beyond loss of function to biasing AIM binding preference. Through biophysical analyses, we confirm impaired binding of the L341V-AIM mutant to LC3A, LC3B, GABARAP, and GABARAPL1. In contrast, p62 AIM interactions with LC3C and GABARAPL2 are unaffected by this mutation. Isothermal titration calorimetry and NMR investigations provided insights into the entropy-driven GABARAPL2/p62 interaction and how the L341V mutation may be tolerated. Competition binding demonstrated reduced association of the L341V-AIM with one hATG8 manifests as a relative increase in association with alternate hATG8s, indicating effective reprogramming of hATG8 selectivity. These data highlight how a single AIM peptide might compete for binding with different hATG8s and suggest that the L341V-AIM mutation may be neomorphic, representative of a disease mechanism that likely extends into other human disorders.

Autophagy is a central pathway for the removal of aggregated or damaged proteins and organelles and is critical to cellular homeostasis. Deregulation of autophagy is recognized as a pathogenic mechanism contributing to a range of neurodegenerative disorders, including amyotrophic lateral sclerosis (ALS) and frontotemporal lobar degeneration (FTLD) (1, 2, 3, 4). These conditions form a disease spectrum (ALS-FTLD) defined primarily by ubiquitinated inclusions of TAR DNA-binding protein-43 kDa in neurones (5). The link between ALS-FTLD and autophagy is reinforced by the identification of disease-associated mutations in genes encoding key components of the autophagy machinery such as autophagy receptors, *e.g.*, *UBQLN2*, *OPTN*, *SQSTM1* (6, 7, 8, 9, 10). Increased numbers of autophagosomes can be observed in histological analyses of ALS patient tissue samples in the absence of mutations, and these aggregates are also characterized by the consistent presence of SQSTM1/p62 (p62) protein (encoded by *SQSTM1*) and ubiquitin as molecular hallmarks of disease (11, 12). Selected ALS-FTLD-associated mutations of the p62 protein map to the conserved autophagy-related 8 (ATG8) interacting motif (AIM) (13), an unstructured region of the multi-domain 440 residue protein (335-DDDWTHLSS-343). The AIM sequence can be generalized to a [W/F/Y]-X_1_-X_2_-[I/L/V] motif and is critical in p62 for binding to the developing phagophore, through interaction with the lipid-anchored human autophagy-related 8 (hATG8) proteins (Fig. 1*A*) (14, 15, 16). Two complementary roles have been proposed for p62 bound to hATG8s on the phagophore: forming a molecular scaffold for the growing autophagosome and the recruitment of ubiquitinated cargo for degradation through its ubiquitin-associated (UBA) domain (17, 18). We have previously analyzed a seemingly benign p62 AIM mutation (L341V) identified in a late-onset ALS patient that affects a key residue in the hATG8 recognition motif (underlined above) (19). The mutation results in a small but quantifiable (3-fold) reduction in binding affinity for microtubule-associated protein 1A/1B light chain 3B (LC3B) and is associated with a defect in p62 protein recruitment into autophagic vesicles in motor neurone-like cells.

**Figure 1:**
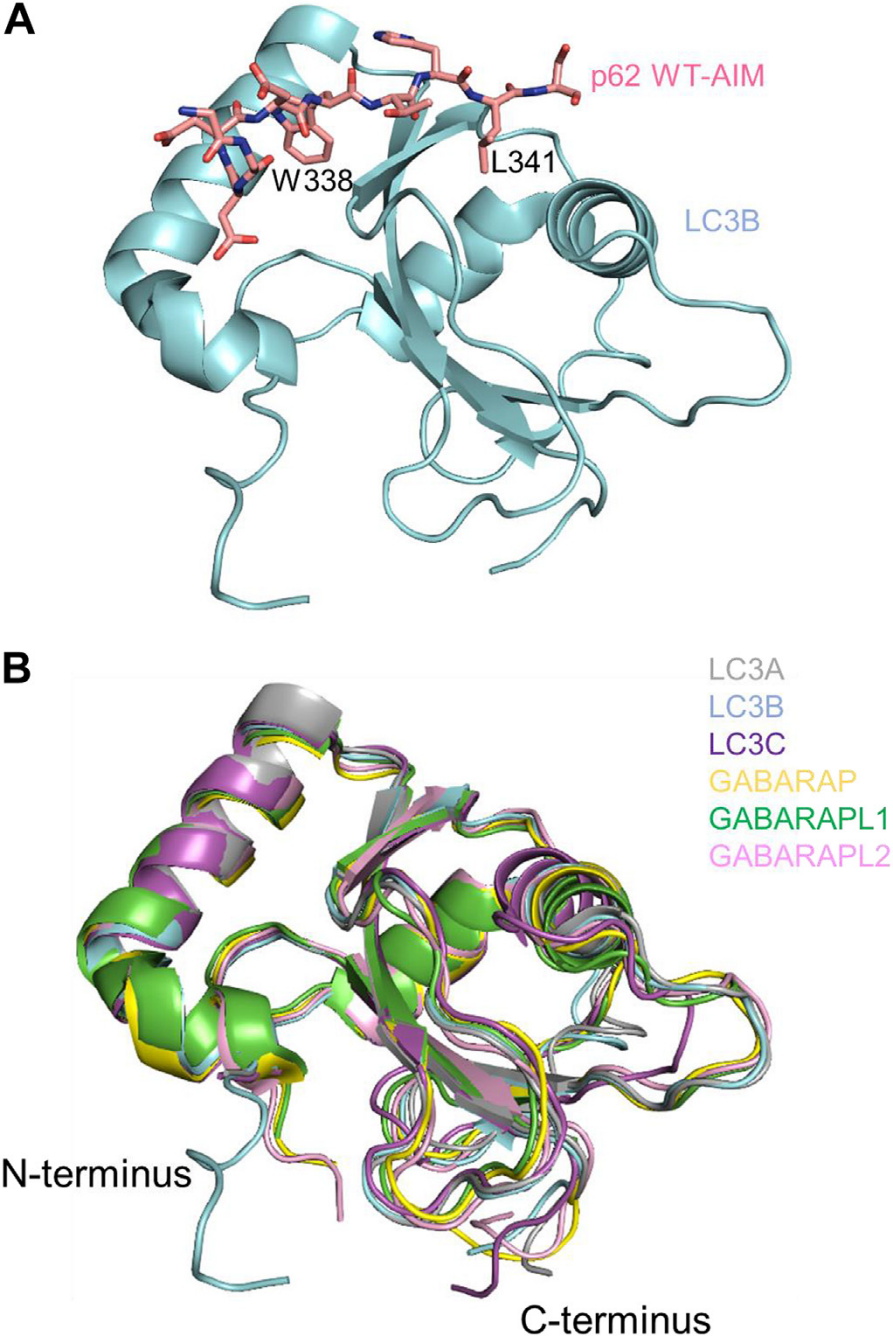
**hATG8 structures and p62 AIM binding.***A,* X-ray structure of LC3B in complex with a p62 WT-AIM peptide (PDB code: 2ZJD) showing key hydrophobic contacts of the W338 and L341 side chains, which are inserted into LC3B surface pockets; (*B*) overlaid structures of the six hATG8 proteins of the LC3 and GABARAP subfamilies showing high structural homology in the overall protein fold, despite significant sequence diversity (PDB codes: 3WAL for LC3A (*gray*), 3VTU for LC3B (*cyan*), 3WAM for LC3C (*magenta*), 1GNU for GABARAP (*yellow*), 2R2Q for GABARAPL1 (*green*), and 4CO7 for GABARAPL2 (*pink*)).

LC3B is one of six hATG8 proteins, which can be divided into two subfamilies: the LC3 subfamily (LC3A, LC3B and LC3C) and the γ-aminobutyric acid receptor associated protein subfamily (GABARAP, GABARAPL1, and GABARAPL2) (20, 21, 22). There is a high level of protein sequence conservation between human LC3A and LC3B (83%) and between GABARAP and GABARAPL1 (87%); however, LC3C and GABARAPL2 are outliers from these subfamilies with much lower conservation. The evolutionary divergence from a single ATG8 in yeast to six human homologues suggests functional diversity, perhaps correlated to sequence diversity. Multiple lines of research, including insights afforded by CRISPR-Cas9 screening and systematic studies of engineered hATG8-deficient human cell lines, show important and sometimes specific roles for hATG8 proteins in the induction of autophagy, the recruitment of autophagic cargo, and the maturation and transport of autophagosomes (23, 24, 25, 26). Different hATG8 expression levels have been observed across cell types and tissues, further indicating the importance of these separate functions in different cellular contexts (27, 28, 29). For example, the movement of autophagosomes to the lysosome appears to be controlled by LC3C and GABARAPL2 phosphorylation specifically (30). Cells lacking GABARAPs, and to a greater extent all hATG8s, accumulate ubiquitin-positive aggregates that are also positive for p62 (26). In SH-SY5Y cells, increased GABARAPL1 expression is induced upon proteasomal inhibition, along with p62, and these proteins have been shown to colocalize in protein aggregates in an ALS mouse model (31, 32).

Despite the sequence diversity (as low as 31% identity between LC3A and GABARAP), the hATG8s display a high level of structural similarity whereby they all adopt an ubiquitin-like fold with an N-terminal α-helical extension (Fig. 1*B*). Functional diversity may therefore be imparted by the specific surface residues of each hATG8 that form their AIM binding sites. This is supported by the range of discovered or designed AIMs, which can produce up to tenfold differences in binding selectivity among the hATG8s (33, 34, 35, 36, 37, 38). Sequence-specific study may provide generalized rules for targeting particular hATG8 surfaces by interaction partners, such as the GABARAP interaction motif (GIM) proposed by Rogov *et al.* (34). The Val position in the [W/F]-[V/I]-X_2_-V GIM motif corresponds to the mutated AIM position in the ALS-associated L341V variant of p62 and implies that the decrease in binding affinity we previously observed for LC3B may impart broader changes in hATG8 selectivity, as the variant now presents a more GIM-like sequence. However, the presence of a Thr in the p62 AIM sequence at the X_1_ position, which is Val or Ile in the GIM, makes this switch to GABARAP-binding preference uncertain and worthy of study in this disease-relevant context.

The observations above paint a picture of cellular mixtures of hATG8 proteins with their various interactions with autophagy receptors in equilibria to control the relevant autophagy processes. We can consider the relative expression levels, along with lipidation rates (needed to anchor them to phagophore membranes and promote avid binding of autophagy receptors), of each of the hATG8 proteins to indicate that forming autophagosomes will have spatiotemporally regulated levels of each hATG8 protein attached to the membrane. These hATG8 proteins then have a degree of selectivity in their interactions with the AIMs of various autophagy receptors and adaptors. Therefore, the mixture of receptors and adapters bound to the membrane (through hATG8s) is likely tightly controlled, and this flux influences the process of autophagosome formation, through maturation, to fusion with the lysosome.

In this wider context, we have investigated the impact of the L341V mutation of the p62 AIM on selectivity in binding to the family of hATG8s. We highlight GABARAPL2 for its unique binding mode with the wild type (WT)-AIM sequence of p62, being able to tolerate the L341V mutation, and investigate this interaction structurally. From this we provide an improved understanding of the impact that AIM mutations have on the regulation of hATG8-mediated autophagy and how this may be integrated in the pathogenic mechanisms of ALS-FTLD and indeed other diseases.

## Results

### ESI-MS interaction profiling reveals differential effects of L341V mutation on p62 AIM peptide binding to hATG8 proteins

To probe hATG8/p62 AIM interaction profiles, electrospray ionization–mass spectrometry (ESI-MS) with optimized nondenaturing conditions (39) was used to study noncovalent interactions between all six purified hATG8 proteins and synthetic p62 AIM peptides, based upon the WT-AIM sequence: SGGDDDWTH**L**SS (with L341V-mutant peptide containing V at the underlined position). We have previously shown that this approach affords a rapid and sensitive method to profile protein–protein interactions in the gas phase, as a convenient alternative to other biophysical techniques (40).

**Figure 2:**
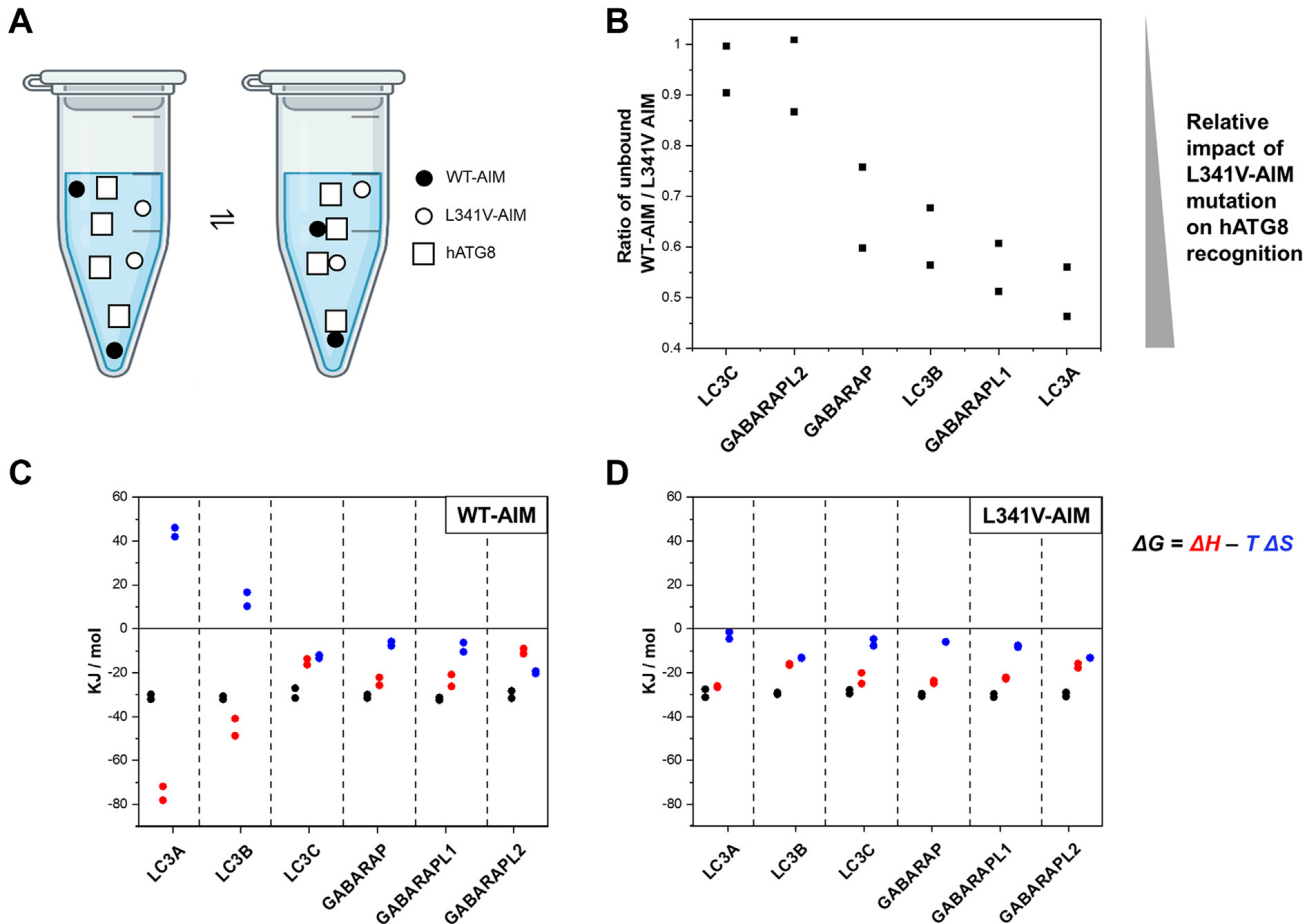
**Effect of the L341V mutation of p62 AIM on hATG8 binding.***A,* cartoon reaction scheme illustrating the equilibrium mixture under investigation. *B,* graphical representation of ESI-MS competition binding assay data for each of the hATG8 proteins with an equimolar mixture of p62 WT-AIM and L341V-AIM peptides, showing the ratio of the unbound peptides upon the addition of indicated hATG8 (20 μM). Unity indicates no preference between the peptides, and lower values indicate a preference for the WT-AIM peptide (loss-of-function with the mutant). hATG8 proteins are ordered by this preference with LC3C and GABARAPL2 showing no selectivity and LC3A showing the largest preference for the WT-AIM peptide over mutant. Datapoints from two independent experiments are presented on the scatter plot. Isothermal titration calorimetry data showing *ΔG*, *ΔH* and *ΔS* profiles for all six hATG8s binding to (*C*) WT-AIM or (*D*) L341V-AIM. Relatively small variations in ΔG are underpinned by considerably larger variations in *ΔH* and *ΔS*, which are altered by the L341V mutation. ESI-MS, electrospray ionization–mass spectrometry.

We first set up an ESI-MS competition binding assay to probe effects of the p62 AIM mutation on hATG8 interactions, in which each of the six hATG8 proteins was individually titrated into an equimolar mixture of WT-AIM and L341V-AIM peptides (each AIM peptide at 10 μM). The unbound AIM peptides were detected in two charge states ([M + 1H]^+^ and [M + 2H]^2+^), and unbound hATG8 could be detected at various charge states (ranging from [M + 6H]^6+^ to [M + 11H]^11+^ depending on the protein); however, the two bound hATG8/AIM complexes were not resolved due to the small difference in *m/z* caused by smaller differences in *m/z* at higher charge states, *z*. Binding preference for a given hATG8 was therefore monitored using the ratio of the peak intensities of the WT and mutant unbound AIM peptides, at *m/z* 1277 and 639 for the WT peptide, or 1263 and 632 for the L341V peptide, with a ratio <1 for WT:L341V indicative of preferential binding of hATG8 to the WT-AIM (example spectra for GABARAP, ratio 0.68, in Fig. S1). This methodology was implemented across all six hATG8s and concentration dependency curves generated from averaging two independent experiments. The ratios for the 20 μM hATG8 data points (1:1 hATG8:peptide mixture) produced the clearest differences in selectivity, indicating a range of binding preferences, with GABARAP, LC3B, GABARAPL1, and LC3A showing varying degrees of preference for the WT-AIM peptide compared with mutant, with the unbound WT:L341V AIM ratio dropping to ∼0.5 for GABARAPL1 and LC3A, implying strong preference for WT-AIM (Fig. 2, *A* and *B*). In contrast, LC3C and GABARAPL2 showed little selectivity (ratios >0.95) under identical experimental conditions, indicating mutation of the p62 AIM has minimal impact on binding affinity of these hATG8s. Consistent with our previous observations, LC3B showed clear preference in binding the WT-AIM peptide over the L341V mutant (unbound WT:L341V ratio ∼0.6) (19).

### Diverse energetic contributions account for the L341V-AIM mutation-associated alterations in hATG8 affinity

We used isothermal titration calorimetry (ITC) to provide corroborating quantitative data on the general trends seen by ESI-MS. We first determined binding affinities and enthalpies of interaction for the WT-AIM peptide with each of the hATG8s (Figs. 2*C* and S2, Table 1), giving *K*_*D*_ values in the range 3.23–16.84 μM. LC3C produced the weakest interactions, and GABARAPL1 and LC3B the strongest, representing a fivefold range of affinities. Overall, the low micromolar affinities we measured are entirely consistent with previously reported data (19, 34). Strikingly, the differences in the enthalpic/entropic contributions are considerably more variable than the affinities, with LC3A and LC3B showing negative entropic contributions counteracted by a strong enthalpy contribution, for example, differing from other hATG8 proteins. This is consistent with strong *van der Waals* packing between side chains within the separate surface binding pockets for the W338 and L341 residues of the WT-AIM peptide (SGGDDD**W**TH**L**SS) as observed in the crystal structure of LC3B/WT-AIM (13). In contrast, GABARAPL2, which shows the second lowest affinity for WT-AIM, demonstrates a unique entropy-driven binding mode. The remaining hATG8s exhibit enthalpically driven interactions with small entropic components, to varying degrees to produce their moderate range of affinities.

**Table 1:** ITC-derived thermodynamic parameters for the binding of WT-AIM or L341V-AIM to LC3 and GABARAP hATG8 proteins

hATG8	*Stoichiometry, n*	*K*_*D*_ (μM)	*ΔH* (kJ mol^−1^)
WT-AIM			
LC3A	1.05 ± 0.02	4.55 ± 0.30	−74.97 ± 1.57
LC3B	0.92 ± 0.03	3.83 ± 0.41	−44.85 ± 1.83
LC3C	0.82 ± 0.13	16.84 ± 4.08	−15.01 ± 3.52
GABARAP	0.92 ± 0.01	5.20 ± 0.31	−23.97 ± 0.34
GABARAPL1	0.86 ± 0.02	3.23 ± 0.45	−23.49 ± 0.86
GABARAPL2	1.07 ± 0.06	6.85 ± 1.02	−10.15 ± 0.69
L341V-AIM			
LC3A	0.87 ± 0.08	8.70 ± 1.84	−26.37 ± 3.50
LC3B	1.14 ± 0.01	8.47 ± 0.61	−16.20 ± 0.33
LC3C	0.90 ± 0.02	11.40 ± 0.95	−22.52 ± 0.98
GABARAP	0.87 ± 0.02	6.41 ± 0.52	−24.19 ± 0.87
GABARAPL1	0.98 ± 0.02	5.78 ± 0.50	−22.46 ± 0.74
GABARAPL2	1.03 ± 0.04	6.99 ± 0.94	−16.75 ± 0.98

We subsequently investigated the binding of the mutant L341V-AIM to the six hATG8s under identical ITC conditions as for the WT-AIM (Figs. 2*D* and S3, Table 1). This data supports the trends observed *via* ESI-MS profiling (above), with a reduction in affinity for LC3A, LC3B, GABARAP, and GABARAPL1 and minimal change in affinity for LC3C and GABARAPL2. The three hATG8s with the strongest binding to the WT-AIM peptide experience an approximately twofold reduction in affinity with the L341V mutant, with an average 1.5-fold reduction in affinity across the hATG8 proteins. As the strongest interactions are weakened and the weakest remain relatively unchanged, we observe a much narrower range of binding affinities (twofold range) with less pronounced variation in the thermodynamics of the interaction for the L341V-AIM mutant. The much weaker enthalpic binding component for LC3A and LC3B suggests alterations in the proposed *van der Waals* packing associated with the replacement of L341 with a β-branched valine residue in the mutant AIM, to produce the loss in binding free energy. Indeed, chemical shift perturbation (CSP) analysis of the LC3B interaction with the L341V-AIM from our previous protein NMR studies shows extensive perturbations around the leucine-binding pocket, consistent with some side chain reorganization to adapt to the change in steric requirements in accommodating the leucine-to-valine substitution in this pocket (19).

### NMR mapping of the WT-AIM interaction surface with GABARAPL2

The unique nature of the entropy-driven interaction of the WT-AIM peptide with GABARAPL2, and toleration of the L341V mutation, warranted further structural investigation using NMR. GABARAPL2 was isotopically ^13^C,^15^N-labeled from bacterial cell cultures grown in supplemented minimal medium. ^1^H-^15^N HSQC data were acquired and initially assigned by comparison to the previously reported assignment by Ma *et al.* (41), which identified 91% of the nonprolyl backbone amide resonances (BMRB accession 18827). We confirmed the assignment using two primary sets of 3D NMR data, namely the combination of HNCO/HN(CA)CO and HNCACB/HN(CO)CACB, following standard assignment methodology to connect neighboring residues (42, 43, 44, 45). There was full agreement with the reported assignments.

**Figure 3:**
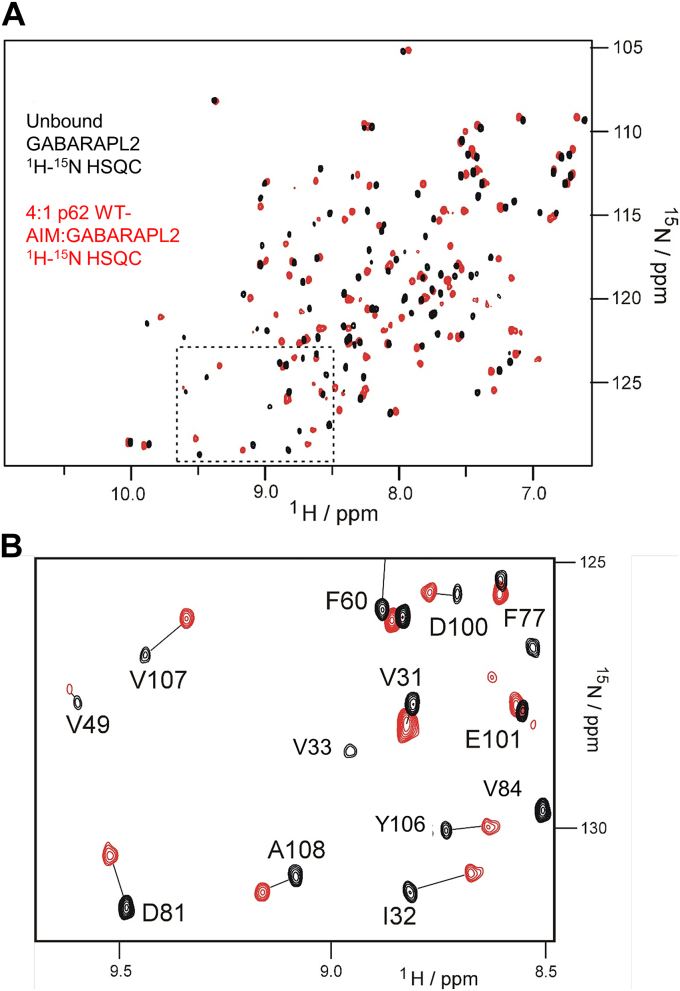
**NMR data showing the CSPs from the WT-AIM titration with GABARAPL2.***A,* overlaid ^1^H-^15^N-HSQC spectra of unbound GABARAPL2 (*black*) and 4:1 complex of p62 WT-AIM bound to GABARAPL2 (*red*), (*B*) an expanded region (*box* in *A*) highlighting CSP between free and bound states. CSP, chemical shift perturbation.

The assigned ^1^H-^15^N-HSQC spectrum of the protein provides a unique residue-specific protein fingerprint with peak positions sensitive to small local perturbations induced by ligand interactions. Analysis of the perturbing effect provides a powerful method of mapping, and then modeling, ligand interaction sites. For ligand titration studies with WT-AIM peptide, GABARAPL2 was ^15^N-labeled, and ^1^H-^15^N HSQC spectra were collected upon incremental addition of the ligand up to a 4:1 ratio of AIM:protein. Overlaid spectra of the bound (4:1) and unbound states demonstrate substantial CSPs (Figs. 3, *A* and *B* and S4). In total, 73% of assigned signals resulted in fast or intermediate exchange between free and bound states allowing changes in position to be readily tracked through the titration. In total, 28 assigned peaks exhibited slow exchange kinetics associated with larger CSP effects and consequently were used in the mapping process as the most significantly shifted upon ligand binding.

**Figure 4:**
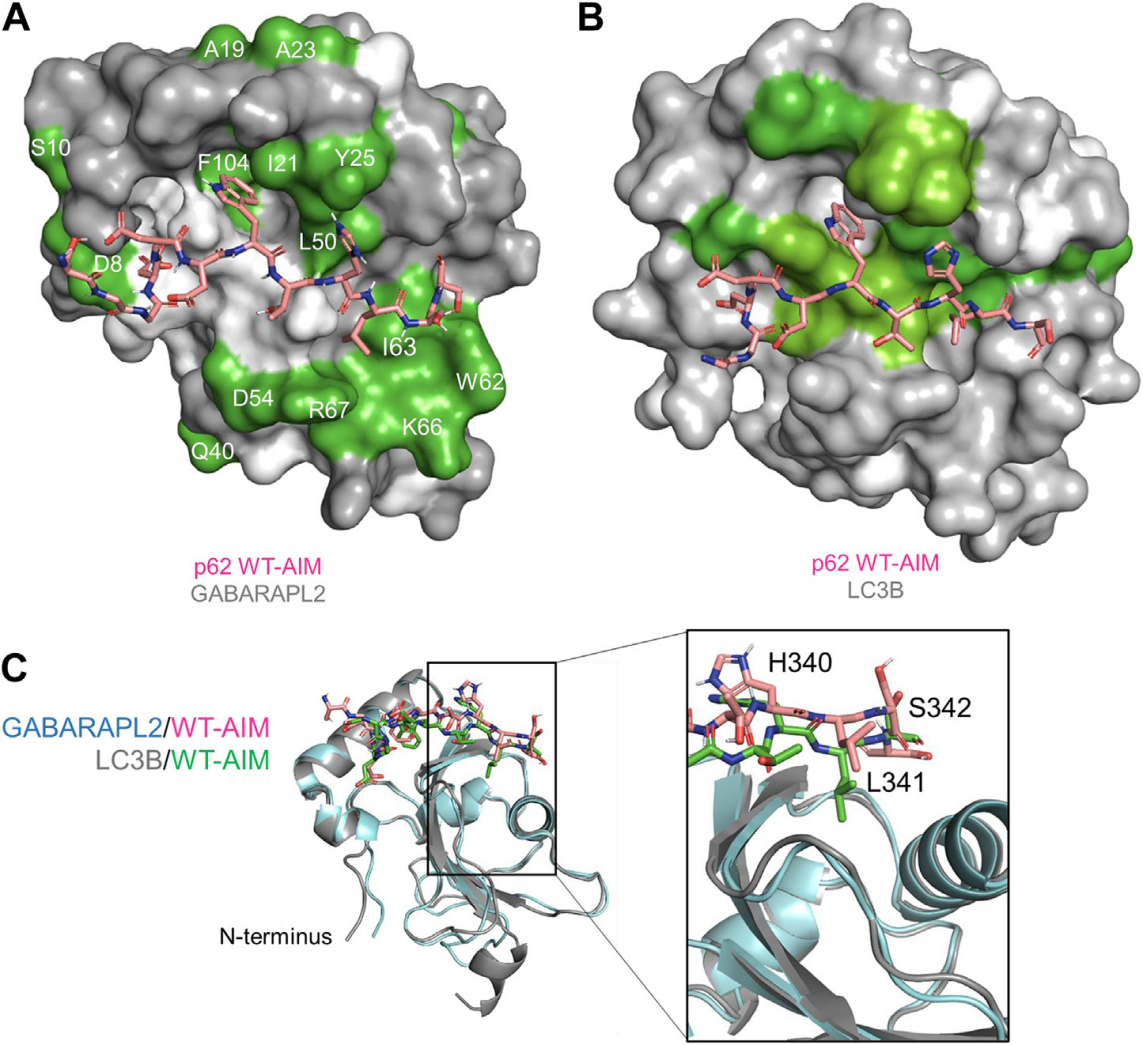
**A modeled structure of p62 WT-AIM bound to GABARAPL2 determined from NMR CSP data, illustrating differences in binding surface with LC3B.***A,* HADDOCK docked structure of the p62 WT-AIM in complex with GABARAPL2 showing the experimentally determined significant NMR CSP effects mapped to the surface. The largest effects, resulting in slow exchange between free and bound, are shown in *green* with residues labelled in *white*; *dark gray* represents unaffected residues and *lighter grey* those that could not be assigned in the unbound state. *B,* CSP for the WT-AIM interaction with LC3B, as previously reported (19) to illustrate similarities and differences in the perturbations around the ligand binding site. A patch of residues identified in the GABARAPL2 complex (D45, W62, I63, K66, and R67) show significant perturbations but have no equivalent in the complex with LC3B, reflecting possible differences in the side chain orientation of L341 of the WT-AIM peptide. *C,* the modelled GABARAPL2/WT-AIM structure is overlaid with the LC3B/WT-AIM crystal structure (PDB code: 2ZJD) to highlight proposed differences in binding mode.

These significantly shifted residues form a binding interface on GABARAPL2, approximately corresponding to the AIM-binding interface observed on LC3B (Fig. 4, *A* and *B*), although a section of the proposed binding surface is unassigned so no CSPs were determined. The W338-binding pocket, analogous to that in LC3B (19), can be observed by the significant CSP of GABARAPL2 residues I21, L50, and F104. However, in the area that would correspond to the L341-binding pocket, there is a much broader patch of significantly shifted residues on GABARAPL2 than was present for LC3B, including D45, W62, I63, K66, and R67. This represents a different mode of binding for the WT-p62 AIM (L341) on the GABARAPL2 surface, where it impacts a larger area of residues. It should be noted that we observe extensive CSPs throughout the protein structure, which are not simply confined to the ligand-binding surface. As with LC3B, we interpret these effects in terms of structural reorganization/tightening of secondary structure interactions that are propagated away from the ligand binding site as part of the induced-fit optimization of the ligand–receptor interaction. These demonstrate that thermodynamic changes on ligand binding reflect holistic effects on ligand and protein structure and not just interfacial contacts. Finally, we constructed binding isotherms from peaks exhibiting fast exchange kinetics, which could be readily followed through the titration and calculated an averaged *K*_*D*_ of 5.2 ± 1.4 μM for the WT-AIM/GABARAPL2 interaction (Fig. S5), which is consistent with our ITC-derived value of 6.85 ± 1.02 μM.

### Modeling the p62 AIM interaction with GABARAPL2

The HADDOCK2.2 (high ambiguity-driven protein–protein docking) webserver (46) was used to calculate a molecular model of the WT-AIM peptide docked to the GABARAPL2 surface based on NMR CSPs, to suggest a structural explanation for the broader binding surface on GABARAPL2 when interacting with the p62 AIM. W338 and L341 were identified as the “active” ligand residues within the AIM, together with the 28 residues on the GABARAPL2 protein surface identified as significant through the NMR mapping (passive residues were automatically defined around the active residues), to produce high-scoring clusters of structures (Supporting Information). The docking was performed three independent times and produced highly structurally similar outputs with an average root-mean-square deviation (RMSD) of 0.61 Å when comparing the lowest energy structures to each other (lowest energy structure shown in Fig. 4*A*). This structure is consistent with other ligand-binding models of hATG8s including the formation of an intermolecular β sheet (13, 47, 48). This model shows W338 binding into a pocket on the GABARAPL2 surface as predicted but produces a novel binding mode for L341. When the X-ray structure of LC3B and the HADDOCK model of GABARAPL2 binding to WT-AIM are both overlaid, there is a striking absence of the hydrophobic leucine binding cleft into GABARAPL2, which is seen in LC3B, along with significant differences in the bound peptide structure (Fig. 4*C*). There is a difference in the packing of the LC3B/GABARAPL2 β2-strand as the LC3B protein accommodates the L341 side chain of the AIM. The L341 side chain of the WT-AIM, of necessity, lies perpendicular to the GABARAPL2 surface, rather than inserted directly into it. This proposed binding mode would rationalize the experimentally observed thermodynamic profile of binding and broader binding surface observed by NMR CSP analysis, which could be experimentally verified in future studies.

### ESI-MS competition experiments reveal a shift in hATG8-binding selectivity associated with the L341V-AIM mutation

**Figure 5:**
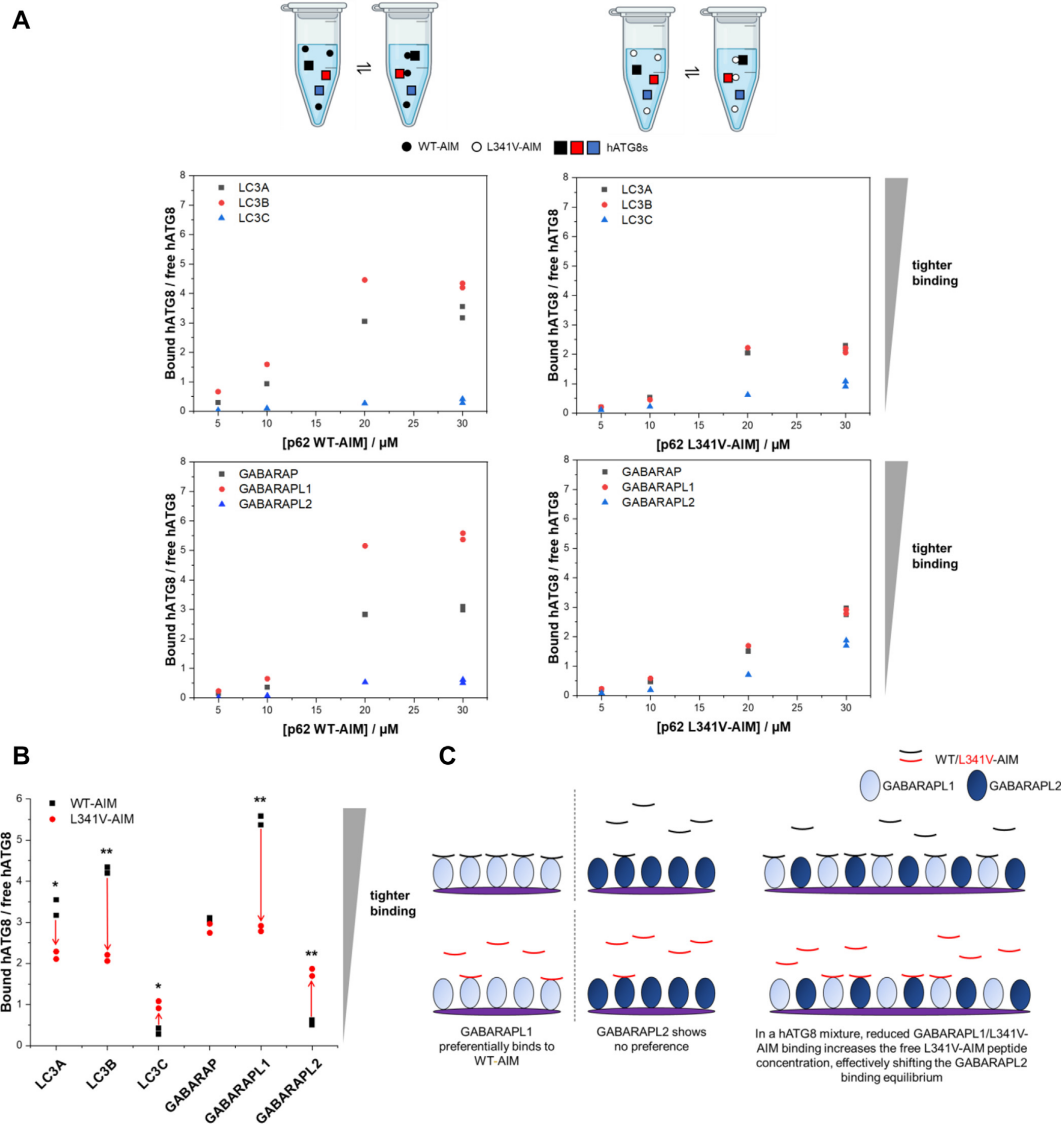
**Effect of the L341V mutation of p62 AIM on hATG8 interactions in the context of mixtures of hATG8 proteins.***A,* data from competition binding experiments showing partitioning of the WT (*left panels*) or L341V (*right panels*) AIM peptide, among the LC3 (*top panels*) and GABARAP (*bottom panels*) subfamilies. Bound:free ratios for each hATG8 at varying peptide concentration are presented to illustrate relative binding affinity (10 μM of each hATG8, making 30 μM peptide equivalent to 1:1 peptide:hATG8s). Data are shown from a single exploratory analysis for the lower concentration points, with data from two independent experiments presented as a scatter for 30 μM (indicating high reproducibility with near overlap of datapoints). *B,* scatter graph showing the bound:free ratio for each hATG8 protein at 30 μM peptide (WT or L341V), with *red arrows* indicating the shifts associated with the L341V mutation. The degree of significance between the WT and L341V measurements, for an individual hATG8, is indicated by *p* values from a *t* test with ∗ indicating *p* ≤ 0.05, and ∗∗ indicating *p* ≤ 0.01. This indicates the L341V mutation is associated with not only an average decrease in affinity, but also reduced hATG8 selectivity. *C,* schematic illustration of the effect of the L341V mutation on binding equilibria to explain these changes in binding preference within hATG8 mixtures. The binding interaction of WT or L341V-AIM peptides to GABARAPL1 or GABARAPL2 is taken as an example with the hATG8 proteins shown on a lipid membrane to indicate the colocalization which occurs *in vivo*. The AIMs would be presented as oligomeric p62 complexes *in vivo*, which is not indicated here. In isolation, GABARAPL1 binds preferentially to the WT-AIM, whereas GABARAPL2 has no preference. When we consider a mixture of GABARAPL1 and GABARAPL2, the increase in unbound L341V-AIM (as a result of impaired GABARAPL1 binding) leads to a shift in the binding equilibria that increases the relative amount of peptide bound to GABARAPL2.

The ITC analyses of p62 AIM/hATG8 interactions (above) provided an important calibration and validation of our ESI-MS profiling approach. Toward modeling p62 AIM interactions in a more “physiological,” phagophore-like environment, we considered AIM selectivity in a mixture of hATG8 proteins, expanding our understanding of the broader effects of the L341V mutation. In these exploratory studies, we adapted the ESI-MS competition binding experiment to probe relative hATG8 binding, by addition of the individual WT-AIM or L341V-AIM peptides into a 1:1:1 equimolar mixture of the three LC3 subfamily members or the GABARAP members (Figs. 5*A* and S6). This divide was necessary as the mixture of all six hATG8 proteins produced a congested mass spectrum with overlapping peaks, not suitable for quantitative analysis (Fig. S7). In these experiments with a single AIM peptide (WT or L341V mutant), we calculate the ratio of bound to free protein for each hATG8 in the mixture, at each peptide concentration, to provide a qualitative assessment of hATG8 preference. Replicate experiments at the highest concentration point of 30 μM (1:1 peptide:hATG8s) confirmed the ESI-MS data to be highly reproducible, with almost overlapping datapoints at this concentration (Fig. 5*B*). By utilizing a *t* test to determine *p* values, a significant shift in bound:free ratios associated with the L341V mutation can be confirmed for all hATG8 proteins except GABARAP. We observed the binding preference of the WT-AIM peptide among the LC3 subfamily (LC3B preferential, with a bound:free ratio of 4.3 at 30 μM peptide) and the GABARAP subfamily (GABARAPL1 preferential, with a bound:free ratio of 5.5 at 30 μM peptide). These data describe the same trends observed in the quantitative ITC and previous ESI-MS competition experiments. For example, at this concentration, GABARAPL1's bound:free ratio of 5.5 for the WT-AIM drops to 2.8 for the mutant peptide (*p* = 0.002). Notably, however, these experiments expose another effect of the L341V-AIM mutation, that is, a “switch” in hATG8 relative binding preference within a mixture of hATG8 proteins that may be of pathophysiological significance. For example, the relative amount of LC3C bound to the mutant peptide, among the LC3 subfamily, increases as a result of the L341V mutation from a bound:free ratio of 0.3 (WT-AIM) to 1.0 (L341V-AIM) at 30 μM peptide (*p* = 0.03, Fig. 5*B*). This is also observed for GABARAPL2 among the GABARAP subfamily, where the ratio increases from 0.6 to 1.8 with the mutation (*p* = 0.007). In that sense, in a “mixed population” of hATG8 proteins anticipated in cellular contexts, reduced association of the L341V-AIM (and its parent mutant autophagy receptor) with one hATG8 protein may manifest as a relative increase in association with a different hATG8, due to a local increase in concentration of “unbound” AIM peptide/protein (Fig. 5*C*). At higher peptide concentrations, the bound state of the GABARAPL2 interaction is more populated as there is more unbound peptide due to the weakening of the GABARAPL1 interaction, for example.

## Discussion

### Ligand selectivity in binding hATG8s

We have presented a detailed biophysical and structural characterization to define the interactions between the p62 AIM and the full family of hATG8 proteins. We extend our previous findings wherein we described the approximately threefold reduction in affinity of ALS-associated L341V mutation of the p62 AIM toward LC3B (19, 49). This further study is crucial given the specific functions of the different hATG8 proteins (24, 36, 37, 38) and demonstrates significant reductions in affinity of the mutant p62 AIM also for LC3A, GABARAP, and GABARAPL1, covering the hATG8s with the tightest interaction with the WT-AIM. Given the recent findings of Sha *et al.* that demonstrated the requirement of a p62/GABARAPL1 axis to clear protein inclusions following proteasomal inhibition (31), it is interesting to speculate that such function may be perturbed by ALS-associated L341V variant with reduced GABARAP1-binding function.

The apparent lack of effect from the L341V-AIM mutation on LC3C and GABARAPL2 binding, the weakest interactions with the WT-AIM, manifests in a reduction of the observed binding selectivity among the hATG8s (Fig. 5, Table 1). Upon AIM mutation, the range of binding affinities among the hATG8 proteins drops as the relative binding affinities of LC3A, LC3B, GABARAP, and GABARAPL1 decrease while the relative binding affinities of LC3C and GABARAPL2 increase, when presented within a hATG8 mixture. This reduced preference of p62 AIM for LC3B or GABARAPL1 may produce an effect comparable to the pathogenic dynamin 2 (DNM2) mutant, which produces defective autophagosome formation by interacting with ITSN1 rather than LC3 (50). This mutant off-target binding results in DNM2 recruitment to the plasma membrane, away from its normal role at the recycling endosome where it binds to LC3B *via* its AIM. In a related manner, we speculate that the ALS-mutant p62 is neomorphic and can more readily interact with LC3C or GABARAPL2 *in cellulo* (as other hATG8 interactions are less efficient), potentially acting as a competitive inhibitor to the “normal” functions of LC3C and GABARAPL2, for example, given that LC3C selectively interacts with NDP52 or GABARAPL2 with UBA5 (37, 38). Thus, this AIM reprogramming and “hATG8 switching” of mutant p62 may add an additional level of autophagic dysregulation through shifts in binding equilibria and autophagy selectivity, in addition to the more direct effects reduction in hATG8 binding will have. This concept has recently been explored in the context of cancer-related AIM mutations (51), where informatic analysis highlighted 222 potential AIM-associated mutations in 148 proteins associated with human cancers but validated only a single AIM mutation and only with respect to “loss of function.” AIM mutations are likely common in human disease, given how common AIM sequences are across the proteome and as such altered autophagy selectivity may be a widespread disease mechanism that has been overlooked to date.

### Distinct binding interactions of hATG8 proteins

Despite the moderate range of binding affinities observed across p62 AIM/hATG8 interactions, there are significant differences in the thermodynamic contributions to these binding energies. These are indicative of distinct binding modes including the strongly enthalpic interaction of LC3A and LC3B with the p62 WT-AIM peptide. This correlates well with the defined tryptophan and leucine-binding pockets observed in the WT-AIM/LC3B crystal structure (13), to produce strong *van der Waals* packing with the negative entropic effect this rigidity induces. The thermodynamic profile for the interaction of GABARAPL2 with the WT-AIM peptide is uniquely entropy-driven. This along with the more extensive patch of significantly shifted residues in the NMR CSP analysis indicates that the L341 of the WT-p62 AIM packs differently on GABARAPL2 than LC3B, in a way that affects more residues. This difference in binding mode is likely to cause the differential effects upon mutation whereby the L341V-AIM interaction with LC3B is weakened, but the affinity with GABARAPL2 is unaffected. The Leu-to-Val switch can be accommodated by the GABARAPL2 surface. HADDOCK docking software was utilized to suggest a structure for this binding interaction, which indicated that the L341 residue of the WT-AIM does not occupy a defined binding pocket but instead rests on the GABARAPL2 surface to produce the extensive patch of residues, which undergo significant CSPs upon binding, leading to a proposed entropy-driven desolvation effect. Our previous study of the interaction of L341V-AIM with LC3B illustrated more significant structural shifts in the LC3B core to accommodate the β-branched valine residue that were proposed to reduce affinity (19). In the case of the GABARAPL2 interaction, there is no sterically demanding binding pocket involved to accommodate this change in amino acid, and we suggest that the valine may simply occupy a related space on the GABARAPL2 surface, resulting in no significant change to the binding affinity. HADDOCK utilized known structures of the components alongside experimentally determined active residues, known to be involved in the binding interaction, to determine ambiguous interaction restraints upon which the docking is based. As with any computational technique, we acknowledge uncertainty about the validity of the structure produced; however, it rationally explains the entropically driven thermodynamics of the interaction and the broader binding surface determined by NMR CSP analysis.

A comparison may be drawn between the GABARAPL2/p62 AIM model produced here and the GABARAPL2/UBA5 AIM structure, the only published structure of an AIM-bound GABARAPL2 (38). Both involve the formation of an intermolecular β-sheet between the AIM and the β2 strand of GABARAPL2; however, the UBA5 AIM is atypical, and this produces a noncanonical interaction. The UBA5 AIM sequence (WGIELV) leads to the formation of a binding pocket not observed in the GABARAPL2/p62 AIM model proposed here or in any other hATG8/AIM interaction, due to the spacing of the key residues. The RMSD between these two full structures is 1.860 Å, with the comparison between the GABARAPL2 proteins only giving a value of 1.836 Å. The difference in structure is produced by the difference in spacing within the motifs as the W338 and L341 residues of p62 (WTHL) are two residues closer together than the corresponding W341 and V346 of UBA5. The UBA5 AIM spacing allows the W341 residue to occupy a novel binding pocket on the GABARAPL2 surface while positioning the V346 residue to bind into one of the typical hATG8-binding pockets. In the case of the p62 AIM, this type of packing is not possible as the aromatic and hydrophobic AIM residues are too close together. ITC data from the work of Huber *et al.* (38) indicate that the GABARAPL2/UBA5 interaction is more enthalpically driven than the interaction with p62 reported here, as the UBA5 packing into two separate and defined hydrophobic pockets is more akin to the LC3B-p62 interaction, which is enthalpically driven.

### Biological implications resulting from *in vivo* avidity effects

**Figure 6:**
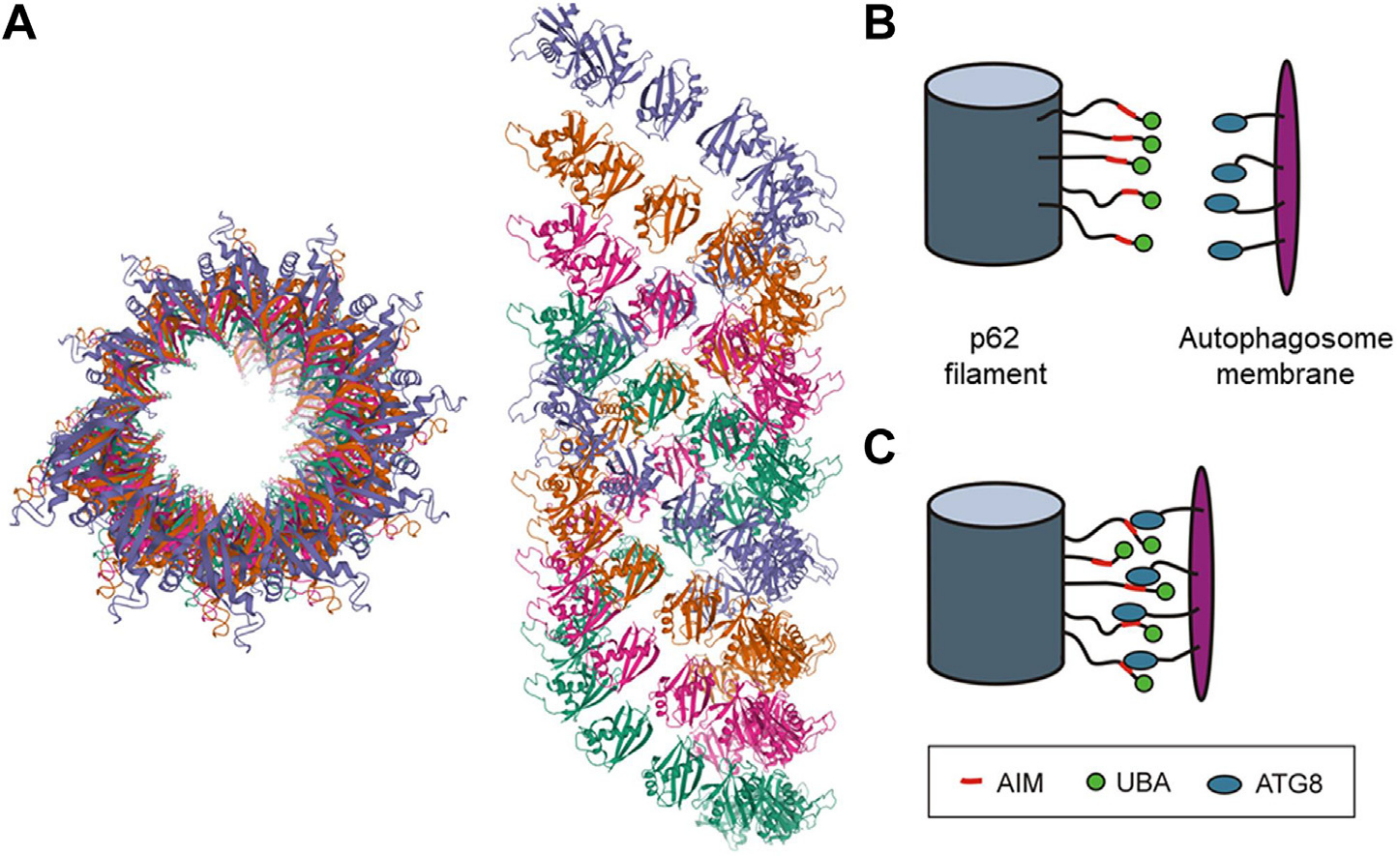
**p62 oligomeric structure promotes avidity effects.***A,* structure of p62 PB1 domains assembled into a cylindrical protein filament shown at two angles, rotated by 90° (PDB code: 4UF8). Of interest are the C-terminal tails that protrude from the PB1 filament, which include the AIM and UBA domains in the full-length protein; (*B*) schematic representation showing the extended p62 C-termini on the filament surface plus lipid-anchored hATG8s in the autophagosome membrane that allow for avid interactions between the two multidentate surfaces (*C*), which come together like “molecular velcro.” Modifications that perturb individual AIM/hATG8 interactions are potentially amplified in the context of these multidentate surfaces.

A clear effect of the ALS-associated L341V mutation of p62/hATG8 interactions has been shown through these *in vitro* studies, though the impact remains relatively modest with individual interaction affinity alterations of up to approximately twofold. However, the interactions are best contextualized around the tendency for p62 to assemble into nanoscale filaments, which have been amenable to cryo-EM structural characterization (Fig. 6*A*) (17, 52). The N-terminal PB1 domain of p62 assembles into a helical protein scaffold with the flexible C-terminal chain, which carries the AIM and UBA domain, extending away from the filament surface as largely unstructured polypeptide tails. This structure presents multiple interaction sites available for binding hATG8 molecules in close proximity. Similarly, hATG8s are lipid-anchored to the growing phagophore membrane, which presents a multidentate surface on which ligands can interact. A combination of the two “velcro surfaces” presents an attractive model for enhanced binding through the avidity effects of multiple interactions (Fig. 6, *B* and *C*). Avidity effects are common in biology, with the work of Sims *et al.* demonstrating a tenfold increase in the affinity of K63-linked di-ubiquitin for the tandem ubiquitin interacting motifs of Rap80 over the individual domains (53). A further example comes from our own studies of an engineered tandem-ubiquitin binding domain interaction with K48-linked diubiquitin chains (54). The moderate affinity alterations observed in our *in vitro* studies presented with monomeric AIM/hATG8 interactions must therefore be considered in these terms, of amplified avid interactions where they may become even more relevant. We have previously shown that the L341V mutation leads to a reduction in p62 recruitment to acidic autophagic vesicles in motor neurone-like cells (19). These perturbations to the functioning of autophagy, alongside other contributing factors, may then be considered over the lifetime of neurones to promote degeneration and a tendency toward cell death over several decades.

## Experimental procedures

### Protein preparation and plasmids

Plasmid DNA encoding each of the hATG8 proteins was inserted into the pGEX-4T-1 plasmid between the *Bam*HI and *Xho*1 restriction sites for expression with an N-terminal GST-tag. Purified plasmid DNA was sequenced in house using a 3130xl ABI PRISM Genetic Analyzer. Protein overexpression was performed in BL21 (DE3) *E. coli* by IPTG (1 mM in culture) induction before incubation with shaking (20 °C, 180 rpm) overnight. Cells were harvested from the culture by centrifugation (3000*g*, 4 °C) for 30 min. Cell pellets were stored at −80 °C. For ^13^C/^15^N protein labeling, M9 minimal media was used in 1L batches supplemented with d-Glucose-^13^C and Ammonium-^15^N chloride as the sole sources of carbon and nitrogen. Cells were lysed in Cleavage Buffer (10 mM Tris, 150 mM NaCl, 25 mM CaCl_2_, 1 mM DTT, pH 8.4) and then centrifuged (35,000*g*, 4 °C). The supernatant was incubated with Glutathione Sepharose 4B beads (1 h, 4 °C) and then washed with cleavage buffer before the addition of human α-thrombin (15 Units/Liter of culture) and incubated with rotation (overnight, 4 °C, 30 rpm) to remove the protein from the GST-tag. Cleaved protein was eluted by gravity and then further purified on AKTA Prime and AKTA Start systems (GE Healthcare) using a HiTrap SP HP anionic exchange column (GE Healthcare) with 5 mM potassium phosphate buffer (pH 7.5) and a 0–1 M NaCl gradient, followed by a Superdex 75 Gel Filtration column (Amersham Biosciences) with 20 mM Tris, 150 mM NaCl buffer at pH 7.5. Finally, a HiTrap Desalting column (Amersham Biosciences) was used to buffer exchange protein into 25 mM ammonium acetate, which was removed by repeated lyophilization to give pure protein powder. Peptides were purchased from Genosphere Biotechnologies and Peptide Synthetics and had the following sequences: p62 WT-AIM – SGGDDDWTHLSS; p62 L341V-AIM – SGGDDDWTHVSS.

### Biophysical studies

#### Mass spectrometry

Experiments to confirm protein identity and purity were performed under denaturing conditions using a Bruker Ultraflex III Mass Spectrometer using matrix-assisted laser dissociation ionization (MALDI) and a time-of-fight mass analyzer. Samples (1 mg/ml in milliQ) were prepared as a 2:1 Super-DHB matrix:protein mixture. Native MS experiments were performed on a Bruker Impact II Mass Spectrometer using electrospray ionization and a quadrupole time-of-flight (qTOF) mass analyzer operating in positive ion mode. Spectra were acquired between 500 and 3000 *m/z* for 1 min under the following optimized conditions: capillary voltage, 2.5 kV; cone voltage, 40 V; trap CE, 8 V; transfer CE, 5 V; backing pressure, ∼3.8 mbar; trap pressure, 2.1 × 10^−2^.

#### ITC analysis

Protein samples (10 μM) were equilibrated in ITC buffer (25 mM potassium phosphate, 150 mM NaCl, pH 7.0) at 30 °C for 1 h within a MicroCal VP-ITC instrument (Malvern) prior to injection of 10 μl aliquots of peptide stock solution (WT-AIM or L341V-AIM at 100–150 μM), with all experiments carried out in duplicate. Control experiments were also carried out by injection of the peptide sample into ITC buffer alone to determine the heat of dilution, which was subtracted. Despite the presence of free solvent-exposed thiols from cysteine residues of LC3A, LC3C, and GABARAPL2, no reducing agents were used in these samples due to potential issues with baseline alteration. ITC titration data were analyzed using MicroCal Analysis software fitting to a one site binding model (55). Presented values were calculated from two independent measurements. All errors are given to one standard deviation.

#### NMR analysis

Experiments were carried out on a Bruker AV(III) 800 spectrometer (Bruker, UK) with a QCI cryoprobe. Standard Bruker pulse sequences were used at 298 K. 1D solvent suppression was carried out using excitation sculpting or WATERGATE solvent suppression. NMR samples were prepared by resuspension of lyophilized protein in NMR buffer (25 mM potassium phosphate, 20 mM NaCl, 5% (v/v) D_2_O, 0.02% (w/v) sodium azide) before centrifugation (13,000*g*, room temperature) for 10 min. Data processing was carried out using Topspin 3.5, and spectra were then analyzed using CcpNmr Analysis (56). Backbone assignment of GABARAPL2 was carried out using a combination of 2D and 3D experiments (^1^H-^15^N-HSQC, HNCO, HN(CA)CO, HNCA, HNCACB, and HN(CO)CACB) and using pairs of related experiments, in particular HNCO/HN(CA)-CO and HNCACB/HN(CO)CACB using established protocols (42, 43, 44, 45). Titration experiments with unlabelled peptides were conducted at 298 K by collecting ^1^H-^15^N-HSQC spectra on ^15^N-GABARAPL2.

### HADDOCK modeling

The HADDOCK2.2 webserver (46) was utilized to model the complex of p62 WT-AIM and GABARAPL2. The active residues on GABARAPL2 were defined from the NMR CSP experiments, and the W338 and L341 residues were defined as active for the WT-AIM p62 peptide. This calculates a HADDOCK score as a weighted sum of electrostatic, van der Waals and restraint energy values. The webserver also provides clustering analysis, determined by grouping at least four structures with common contacts.

### UniProt accession IDs

SQSTM1 Q13501

MAP1LC3A Q9H492

MAP1LC3B Q9GZQ8

MAP1LC3C Q9BXW4

GABARAP 095166

GABARAPL1 Q9H0R8

GABARAPL2 P60520

## Data availability

All data are contained within the manuscript and Supporting Information.

## Supporting information

This article contains supporting information.

## Conflict of interest

The authors have no competing financial interests to declare.
