# Supplementary material for: An ALS-associated variant of the autophagy receptor SQSTM1/p62 reprograms binding selectivity toward the autophagy-related hATG8 proteins
Source: J Biol Chem. 2021 Dec 18;298(2):101514. doi: 10.1016/j.jbc.2021.101514 (PMC8762078; doi:10.1016/j.jbc.2021.101514)
Supplement: Supporting information [file mmc1.docx]

**
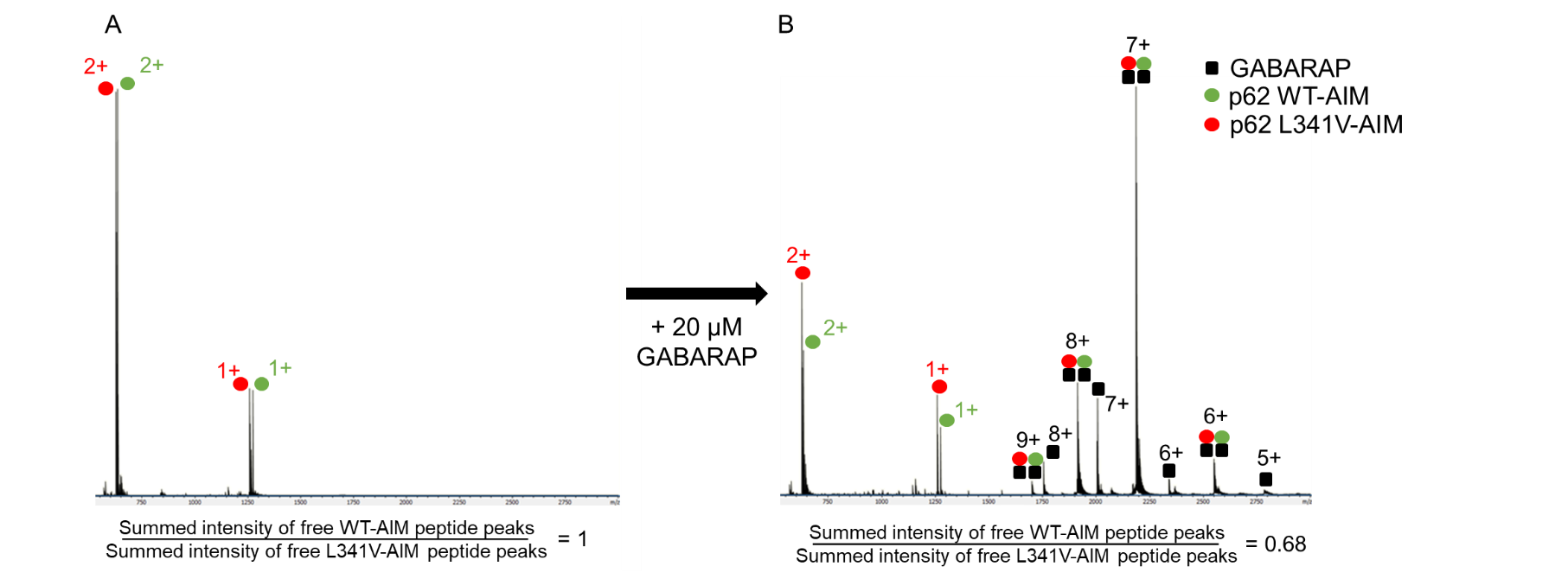
**

**S1 – ESI-MS spectra showing a competition binding experiment with the p62 WT-AIM and L341V-AIM peptides interacting with GABARAP, illustrating calculation used to analyse the data and compare between hATG8 proteins.**

(A) The ESI-MS spectrum of an equimolar mixture of p62 WT- and L341V-AIM peptides (10 µM each) shows the peptides in two charge states with equal summed intensities (ratio of unbound WT-AIM/unbound L341V-AIM ~1). (B) The ESI-MS spectrum of GABARAP (20 µM) in an equimolar mixture of p62 WT-AIM and L341V-AIM peptides (10 µM each) shows peaks corresponding to a mixture of free GABARAP protein and the GABARAP/AIM peptide complexes. The peak intensity for the two unbound WT-AIM peptide peaks is lower than for the unbound L341V-AIM peptides peaks upon addition of GABARAP as the WT-AIM peptide is being preferentially bound (ratio of unbound WT-AIM/L341V-AIM = 0.68). Comparison between the intensities of bound GABARAP peaks is not possible as the peaks are unresolved.

**
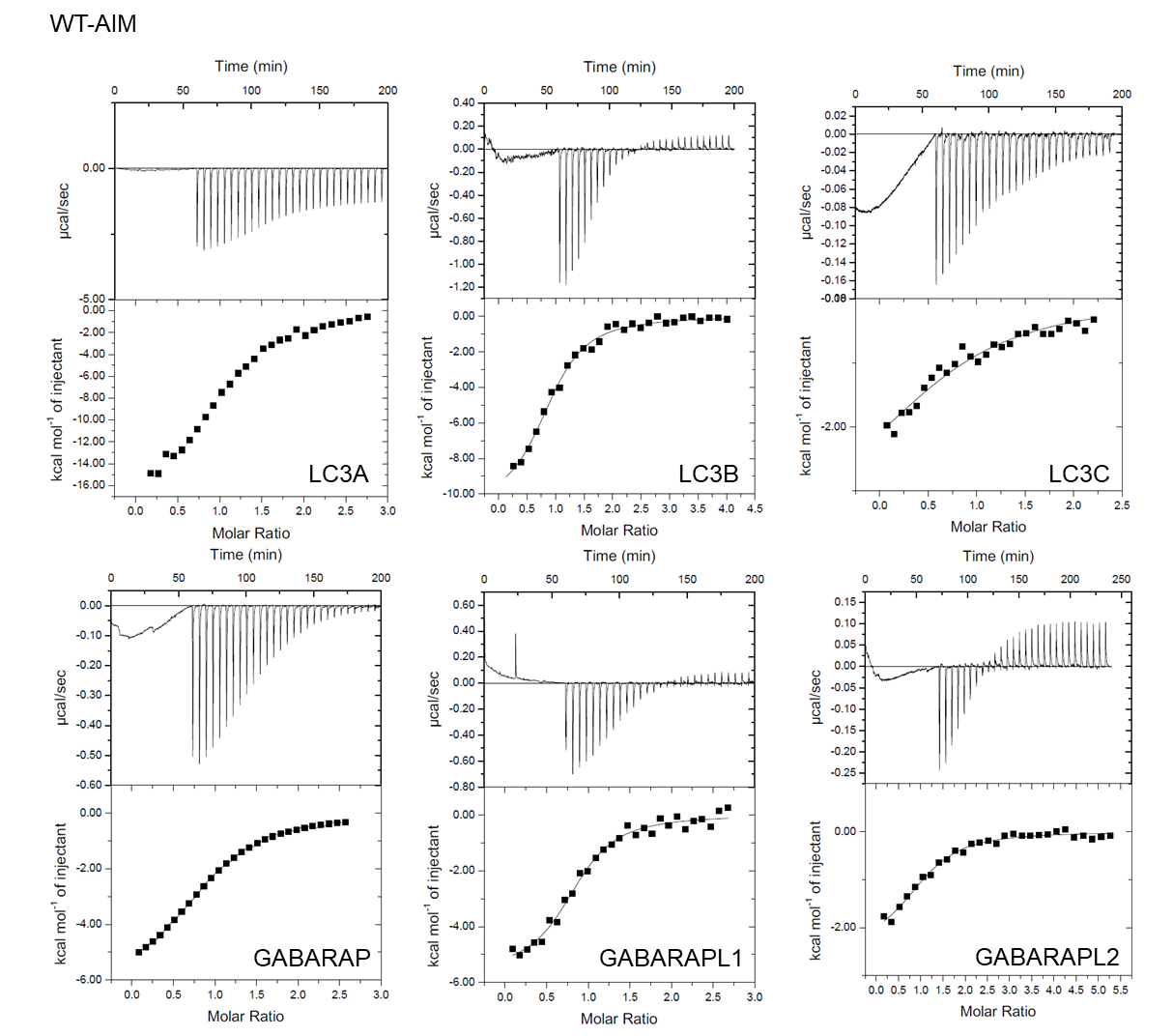
**

**S2 – ITC data showing the interaction of the p62 WT-AIM with the six hATG8 proteins.**

For each hATG8 protein, the raw power compensation plot throughout the titration is shown in the upper graph and the integrated data points and single site model fit (MicroCal ORIGIN software is shown in the lower graph0.


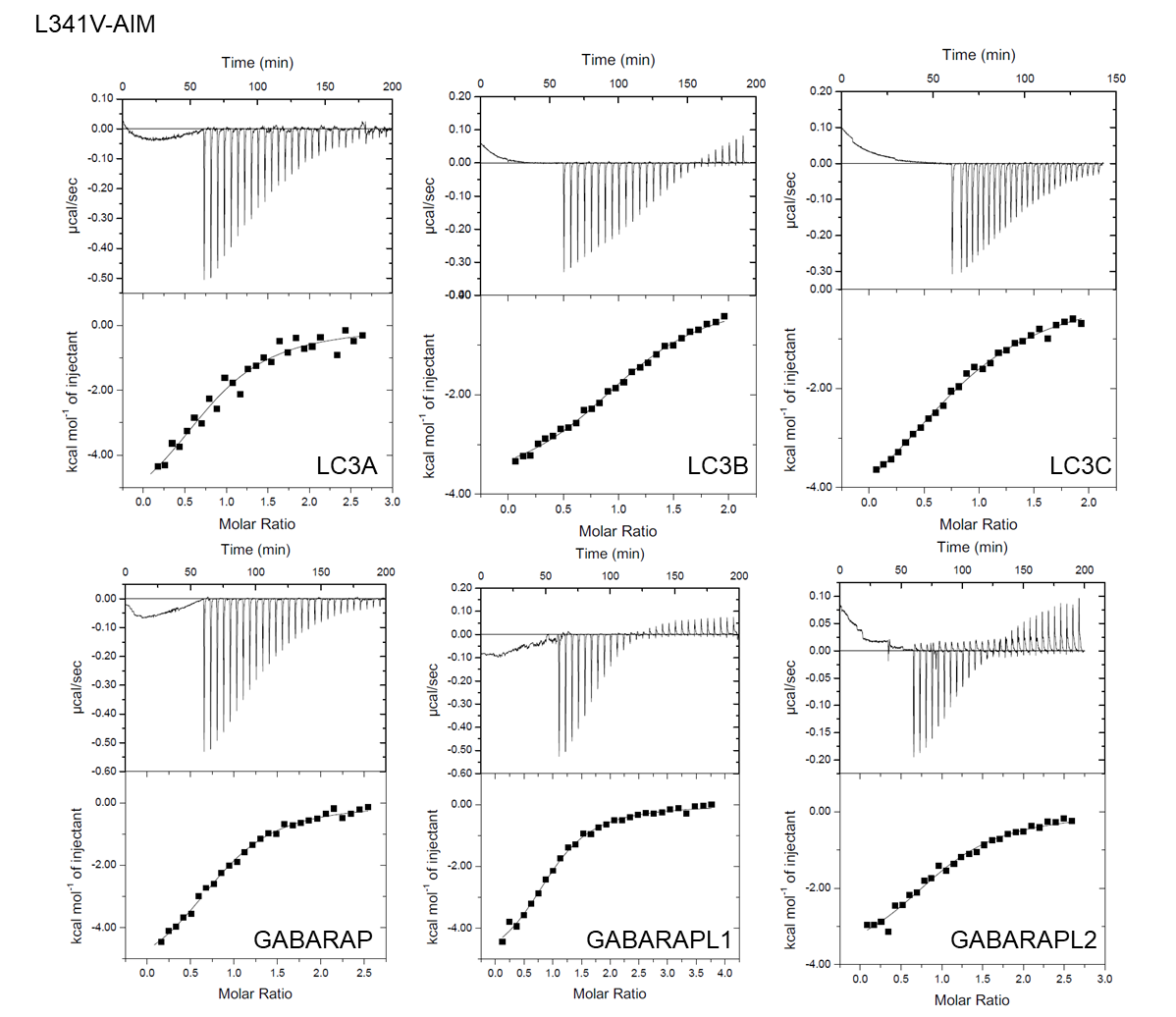


**S3 – ITC data showing the interaction of the p62 L341V-AIM with the six hATG8 proteins.**

For each hATG8 proteins, the raw power compensation plot throughout the titration is shown in the upper graph and the integrated data points and single site model fit (MicroCal ORIGIN software is shown in the lower graph).


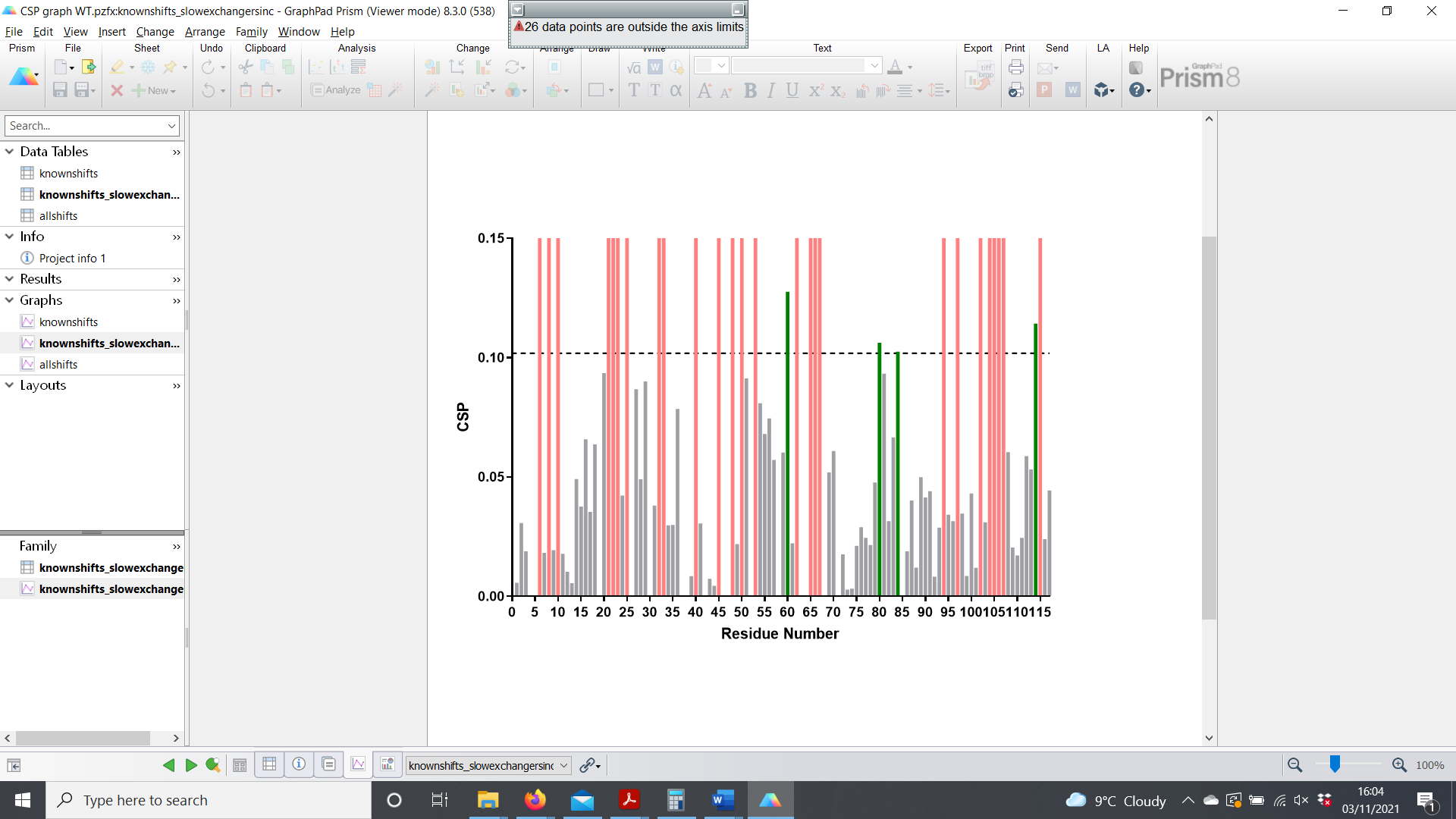


**S4 – CSP perturbation by ^15^N-GABARAPL2 by residue upon p62 WT-AIM binding at a final ratio of 4:1 WT-AIM:GABARAPL2.**

Red bars represent residues with the most significant CSP as they exhibited slow exchange behaviour. The significantly shifted residues exhibiting fast exchange behaviour were defined as two standard deviations above average (dotted line) and the bars of these residues are shown in green.

**S5 – Fitting of the ^15^N-GABARAPL2 CSP data for the four significantly shifted fast exchange residues to determine a *K_D_* value of** 5.2±1.4 µM for binding to the p62 WT-AIM**.**


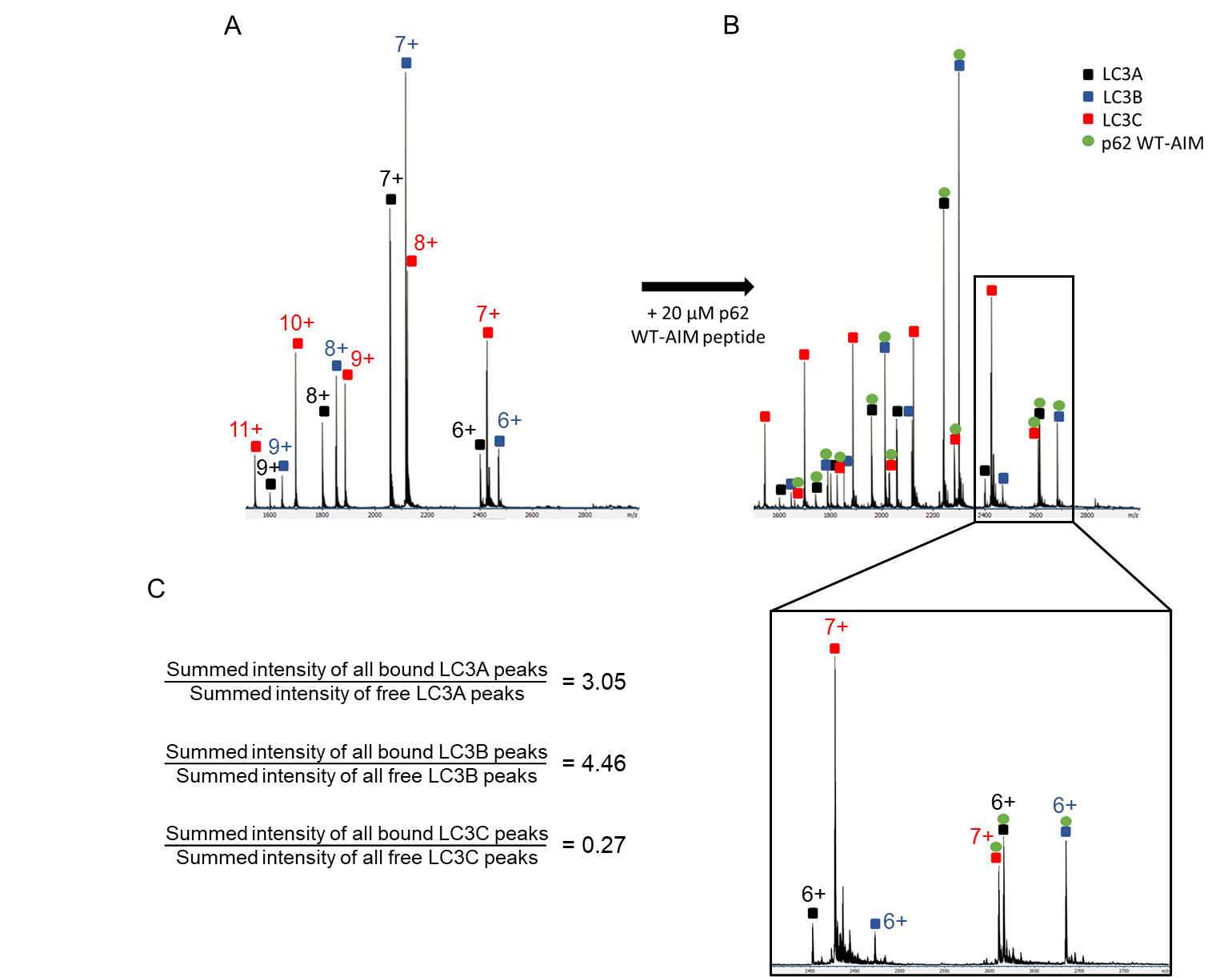


**S6 – ESI-MS spectra showing a competition binding experiment for the LC3 subfamily proteins and the p62 WT-AIM peptide, illustrating the bound/free calculation.**

(A) ESI-MS spectrum of an equimolar mixture of the three LC3 proteins (10 µM each), showing peaks in a range of charge states. (B) ESI-MS spectrum of the p62 WT-AIM peptide (20 µM) added to the LC3 proteins (10 µM each). This spectrum contains a mixture of peaks corresponding to the free or peptide-bound proteins, with a highlighted section showing the LC3A and LC3B [M + 6H]^6+^ ion peaks for both the free and peptide/protein complexes and the LC3C [M + 7H]^7+^ ion peak for the free and peptide/protein complex. (C) The ratios for all the peak intensities corresponding to a bound protein can be summed and divided by the sum of the intensities of the free protein to give a measure of peptide binding in the mixture. This shows that LC3B is the preferred binding partner of the WT-AIM in this mixture and also indicates that most LC3C remains unbound.


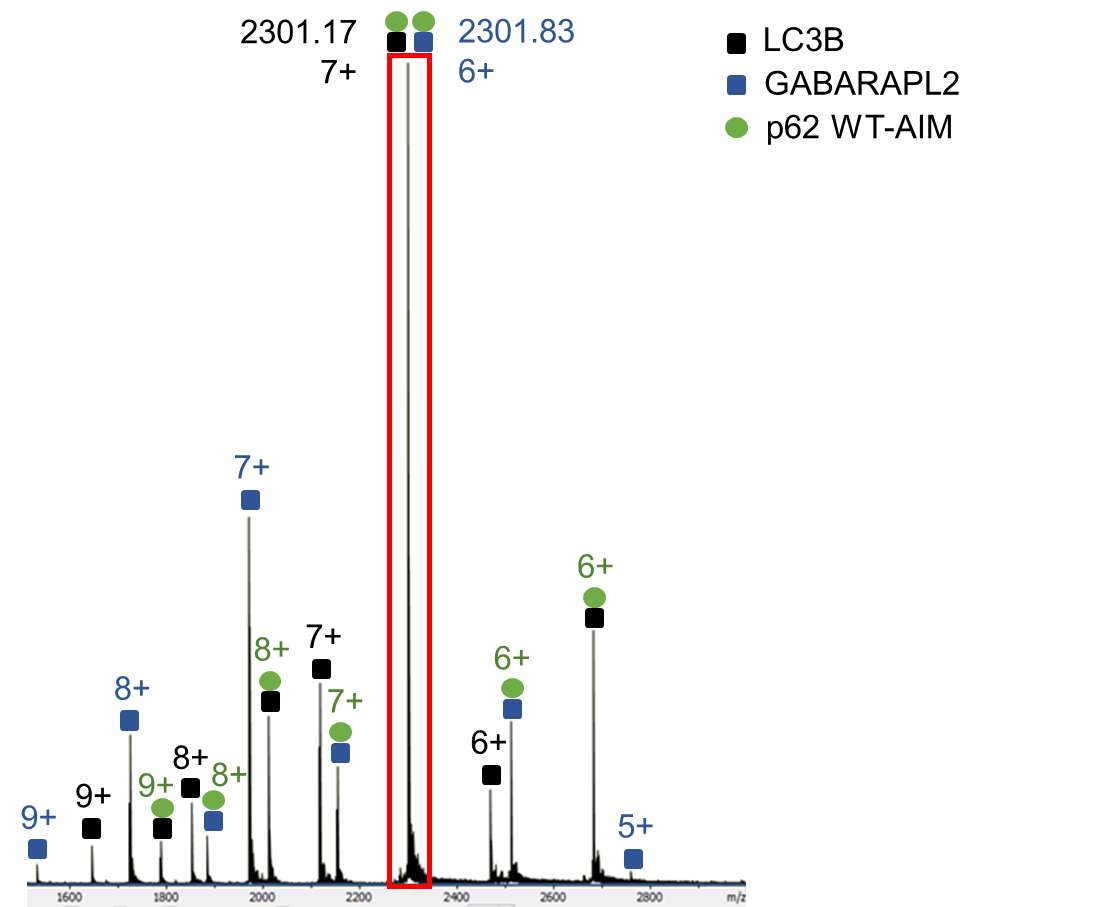


**S7 – ESI-MS spectrum to illustrate an example of overlapping peaks in competition binding experiments, preventing a full panel of hATG8 proteins from being studied simultaneously.**

ESI-MS spectrum of an equimolar mixture of LC3B, GABARAPL2 and the p62 WT-AIM peptide (all at 10 µM). The peak framed by a red box corresponds to two separate species (GABARAPL2 6+ and LC3B/WT-AIM 7+) which occur at similar m/z values and their intensity therefore appears summed. This is the clearest example of peak overlap which prevented quantitative analysis of all six hATG8 proteins in a single experiment and necessitated splitting into two separate competition experiments for the LC3 and GABARAP subfamilies, though other overlaps also existed.

Haddock run 1: HADDOCK clustered **98** structures in **13** cluster(s), which represents **49 %** of the water-refined models HADDOCK generated.

Highest scoring cluster:

| HADDOCK score | -102.6 +/- 7.0 |
| --- | --- |
| Cluster size | 21 |
| RMSD from the overall lowest-energy structure | 1.3 +/- 0.3 |
| Van der Waals energy | -52.3 +/- 6.0 |
| Electrostatic energy | -246.0 +/- 34.3 |
| Desolvation energy | -8.3 +/- 3.2 |
| Restraints violation energy | 52.3 +/- 34.3 |
| Buried Surface Area | 945.3 +/- 60.6 |
| Z-Score | -1.0 |

Haddock run 2: HADDOCK clustered **95** structures in **10** cluster(s), which represents **47 %** of the water-refined models HADDOCK generated.

Highest scoring cluster:

| HADDOCK score | -105.0 +/- 3.1 |
| --- | --- |
| Cluster size | 10 |
| RMSD from the overall lowest-energy structure | 0.7 +/- 0.5 |
| Van der Waals energy | -53.8 +/- 7.3 |
| Electrostatic energy | -300.8 +/- 60.4 |
| Desolvation energy | -1.7 +/- 4.4 |
| Restraints violation energy | 46.2 +/- 28.9 |
| Buried Surface Area | 948.4 +/- 24.3 |
| Z-Score | -1.7 |

Haddock run 3: HADDOCK clustered **101** structures in **9** cluster(s), which represents **50 %** of the water-refined models HADDOCK generated.

Highest scoring cluster:

| HADDOCK score | -92.4 +/- 3.7 |
| --- | --- |
| Cluster size | 22 |
| RMSD from the overall lowest-energy structure | 1.4 +/- 0.5 |
| Van der Waals energy | -48.8 +/- 1.9 |
| Electrostatic energy | -260.8 +/- 7.4 |
| Desolvation energy | -11.2 +/- 2.1 |
| Restraints violation energy | 30.2 +/- 2.4 |
| Buried Surface Area | 890.7 +/- 47.4 |
| Z-Score | -1.8 |
